# High-Capacity Image Steganography Based on Improved Xception

**DOI:** 10.3390/s20247253

**Published:** 2020-12-17

**Authors:** Xintao Duan, Mengxiao Gou, Nao Liu, Wenxin Wang, Chuan Qin

**Affiliations:** 1 College of Computer and Information Engineering, Henan Normal University, Xinxiang 453007, China; gou5695761@163.com (M.G.); liunao18437982698@126.com (N.L.); wangwenxin3023@126.com (W.W.); 2 Laboratory of Artificial Intelligence and Personalized Learning in Education of Henan Province, Xinxiang 453007, China; 3 School of Optical-Electrical and Computer Engineering, University of Shanghai for Science and Technology, Shanghai 200093, China; qin@usst.edu.cn

**Keywords:** image steganography, deep separable convolutional neural network, deep learning, Xception, ResNet

## Abstract

The traditional cover modification steganography method only has low steganography ability. We propose a steganography method based on the convolutional neural network architecture (Xception) of deep separable convolutional layers in order to solve this problem. The Xception architecture is used for image steganography for the first time, which not only increases the width of the network, but also improves the adaptability of network expansion, and adds different receiving fields to carry out multi-scale information in it. By introducing jump connections, we solved the problems of gradient dissipation and gradient descent in the Xception architecture. After cascading the secret image and the mask image, high-quality images can be reconstructed through the network, which greatly improves the speed of steganography. When hiding, only the secret image and the cover image are cascaded, and then the secret image can be embedded in the cover image through the hidden network in order to obtain the secret image. After extraction, the secret image can be reconstructed by bypassing the secret image through the extraction network. The results show that the results that are obtained by our model have high peak signal-to-noise ratio (PSNR) and structural similarity (SSIM), and the average high load capacity is 23.96 bpp (bit per pixel), thus realizing large-capacity image steganography surgery.

## 1. Introduction

Information globalization has become an inevitable trend in the context of the rapid development of computer technology. The same security issues of sensitive information are bound to face huge challenges, and information security is very important. For example, personal private information, medical patient information, commercial business data, military secrets, etc., will have serious consequences if they are leaked. Information security measures are constantly being strengthened in the face of such challenges.

In the early days of information security, encryption technology was used to protect the security of information. Encryption technology makes secret information incomprehensible, but it is also prone to suspicion. Image steganography hides one image in another image in order to make the secret information invisible, thus protecting the communication channel and hiding the communication behavior. The most commonly used cover in information hiding is images. The reasons are as follows: (1) in the context of information technology, images will flow into the Internet on a large scale and be stored. This can provide a large number of samples for information hiding. (2) When compared with text and audio, pictures have more data storage capacity and greater data redundancy, which makes information hiding easier to hide and extract secret information that needs to be protected.

As one of the important methods of image information security protection, image steganography aims to hide sensitive images, which is, secret images, in its irrelevant cover data, and does not affect the cover’s sensory experience, so as to hide information without causing more attention and awareness. Traditional information hiding is based on the continuous improvement of the STC framework [1]. For example, HUGO steganography with a high degree of undetectability [2], Wavelet-Obtained Weights (WOW) steganography [3], JEPG general wavelet relative distortion (J-UNIWARD) steganography [4], a steganography of concatenated codes composed of Syntrrome-Trellis codes (STC), and cyclic redundancy codes (CRC) codes [5], and the more novel scheme [6] uses the decentralized and fair hierarchical threshold secret sharing scheme of blockchain to divide the secret into several shares and assign it to different levels of participants; any small subset can get the secret. There are some shortcomings that require experts in this area to manually design the cost function, which requires attention to the fitting problem while also minimizing the loss; although this works, it is very troublesome. Therefore, using deep learning to design the cost function of steganography has become a novel and reliable steganography scheme. Meanwhile, He et al. [7] proposed a residual learning framework in order to simplify network training deeper than previously used networks and solve the problem of difficulty in training networks with larger depths, indicating that these residual networks are easier to optimize. Additionally, the accuracy can be obtained by increasing the depth. This scheme greatly promotes the wide range of use of neural networks in steganography, and it can maintain a certain accuracy. At present, image steganography that is based on deep learning mainly includes three aspects: image steganography based on Convolutional Neural Network (CNN) [8], image based on Generative Adversarial Networks (GAN) [9] Steganography, and other network structure. Yang et al. [10] proposed a 32-layer convolutional neural network (CNN) in order to improve the efficiency of preprocessing and reusing features by cascading all the features in the previous layers with the same feature map size, thereby improving Information flow and gradient can further reduce detection errors. Chen et al. [11] introduced advanced ideas, such as skip connection and feature fusion, and designed a novel two-channel deep hidden network (TDHN). Wu et al. [12] introduced an attention mechanism in steganography to alleviate security issues and increase the load capacity.

In this work, we use an improved [13] framework, but removed the preprocessing network, and replaced the hidden network with our improved deep separable convolutional Xception network [14] in order to achieve the same size as the cover image and the secret image with the same number of channels (three channel color image or one channel gray image) can be perfectly embedded in the cover image with only slight pixel distortion. The secret image can be steganographically written in all bits and channels of the cover image. The experiments show that our proposed scheme not only effectively improves the steganography capacity, but also the peak signal-to-noise ratio (PSNR) [15] and structural similarity (SSIM) [16] are relatively high.

The main contributions of this article are, as follows:

(1) New plan, we use the improved Xception network for the first time in order to realize image steganography by constructing hidden networks and extraction networks.
(2) Improve Xception to speed up convergence, we have added jump connections to the Xception network to increase the retention of high-dimensional features of the image to speed up the convergence rate when the image is hidden.
(3) High capacity and high quality, we use the improved Xcption network in order to extract the high-dimensional features of the secret image, so that the entire secret image is hidden in the cover image, achieving high-capacity steganography.

## 2. Related Knowledge

In this chapter, we mainly introduce the development and characteristics of Inception V1, Inception V2, and Inception V3, the predecessors of Xception. Because Xception is the ultimate Inception, it is another improvement to Inception V3 that was proposed by Google, so this chapter will briefly describe the Inception improvement process in order to establish the foundation for the Xception architecture later.

### 2.1. Inception V1

In the past few years, with the rapid development of deep learning and convolutional networks, the object classification ability of the network has been greatly improved, and the requirements for the performance of the network have also been continuously improved. There are many ways for improving network performance, such as computer hardware upgrades, better data sets, and so on. How to further improve the performance of the network within the same resources has become the biggest problem. The most important method is to increase the number of layers of the network and the number of channels per layer, which is, the depth and width of the network. However, this method will allow us to add a large number of parameters while increasing the depth and width of the network, which results in a greatly increased amount of calculation and easy overfitting. In order to solve this problem, we determine how the Inception architecture can be proposed to approximate the best local sparse structure of the convolutional vision network, and cover its ideas with easy-to-use dense components [17].

**Figure 1:**
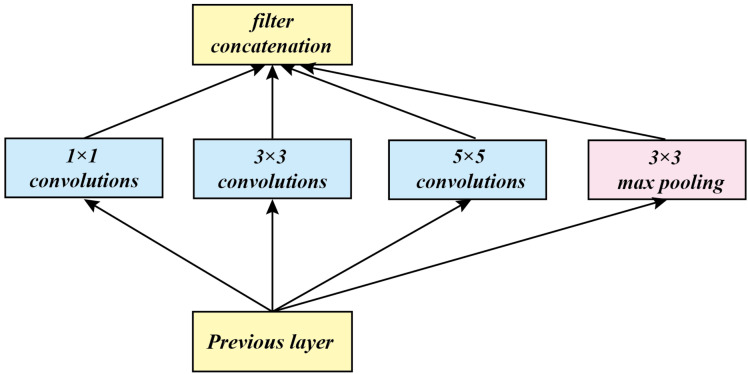
Inception V1 architecture.

The structure of Inception V1 mainly increases the network width, as shown in Figure 1. It provides different receptive fields for each branch, and it finally allows more scale information to stay in it, and the last stitching operation completes different scales. A fusion of features. However, there is a shortcoming of such a network, which is, increasing the width of the network, while increasing the number of parameters, because the number of parameters is too large to cause over-fitting problems. In order to solve this problem, 1 × 1 convolution is introduced to reduce the parameters, and 1 × 1 convolution will introduce more nonlinearity in order to improve generalization ability.

### 2.2. Inception V2

For the Inception V1 network, the setting of initialization parameters and the learning rate is the difficulty of its training. Small changes in the first few layers of the network will be continuously amplified by the subsequent networks. If there are some changes in the input data distribution of a certain layer, then you need to learn this distribution, which results in the network that has been learning new changes to seriously affect the network training speed. Sergey Ioffe et al. [18] proposed normalizing the data. Therefore, Inception V2 introduces BN (Batch Normalization) operations on the basis of Inception V1. The advantages of introducing BN are: (a) a larger learning rate can be set to reduce the network’s dependence on initialization parameters. (b) The problem of gradient dissipation is alleviated, and the original 5 × 5 convolution is replaced with two 3 × 3 convolutions in the Inception V2 framework in order to reduce the number of parameters. The BN operation is to normalize a certain layer, by formula (1):

(1) $$\hat{x^{\left(k\right)}}=\frac{x^{\left(k\right)}-E\left[x^{\left(k\right)}\right]}{\sqrt{Var(x^{\left(k\right)})}}$$

where $E\left[x^{\left(k\right)}\right]$ is the average value of neurons in this batch of data, and $Var(x^{\left(k\right)})$ is the standard deviation of the input value of each neuron. However, if you simply use the normalization operation, then it will have a greater impact on the features learned by the network. Hence, transform reconstruction is proposed, introducing two learning parameters γ and β:

(2) $$y^{\left(k\right)}=\gamma^{\left(k\right)}\hat{x^{\left(k\right)}}+\beta^{\left(k\right)}$$

In this way, each neuron has two corresponding parameters: γ and β. When $\gamma^{\left(k\right)}=\sqrt{Var(x^{\left(k\right)})}$ and $\beta^{\left(k\right)}=E\left[x^{\left(k\right)}\right]$, the features that are learned by the network can be reconstructed.

### 2.3. Inception V3

**Figure 2:**
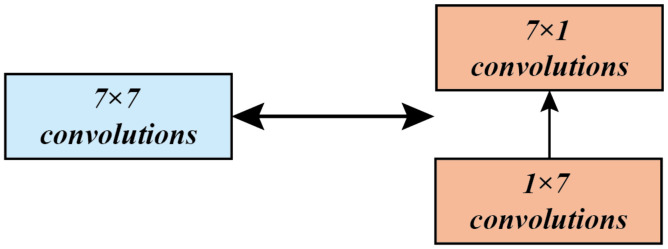
Inception V3’s improvements to Inception V2.

Before we can see that most of the architectures of Inception V1 and Inception V2 use convolution kernels of 3 × 3 or 5 × 5 convolution operations, because, the larger the convolution kernel, the larger the number of network parameters. Accordingly, how to use the convolution operation of a larger convolution kernel with limited resources? Inception V3 proposes decomposing a convolution operation with a kernel size of n × n into a set of convolution operations with a kernel size of 1 × n and n × 1, as shown in Figure 2. The experiments show that this idea can achieve the purpose of deepening the network, accelerating the calculation, and increasing the nonlinearity of the network.

### 2.4. Image Steganography

**Figure 3:**
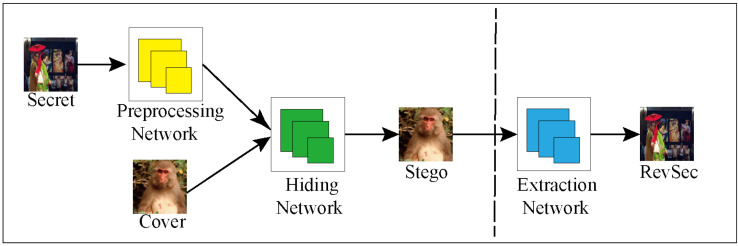
Image steganography architecture.

Baluja et al. [13] proposed the framework for hiding the image in the image. It consists of three parts of the network: preprocessing network, hiding network, and extraction network. Figure 3 shows the general framework. First, the first part of the preprocessing network adjusts images of different input sizes to a uniform size of N × N images (in this paper, the size is 256 × 256); then, the second part of the hidden network is to cascade the adjusted images of the preprocessing network. The output after operation is used as input. The features of the secret image are hidden in the various channels of the cover image and the stego image is generated as similar as possible to the cover image; the third part of the extraction network is to use the output of the hidden network as input from the stego image to extract the hidden secret image.

## 3. Proposed Solution

In this chapter, we mainly describe the specific framework of our proposed solution, the specific content of each part, namely the network architecture, and the composition and function of each small part of the network.

### 3.1. Network Architecture

**Figure 4:**
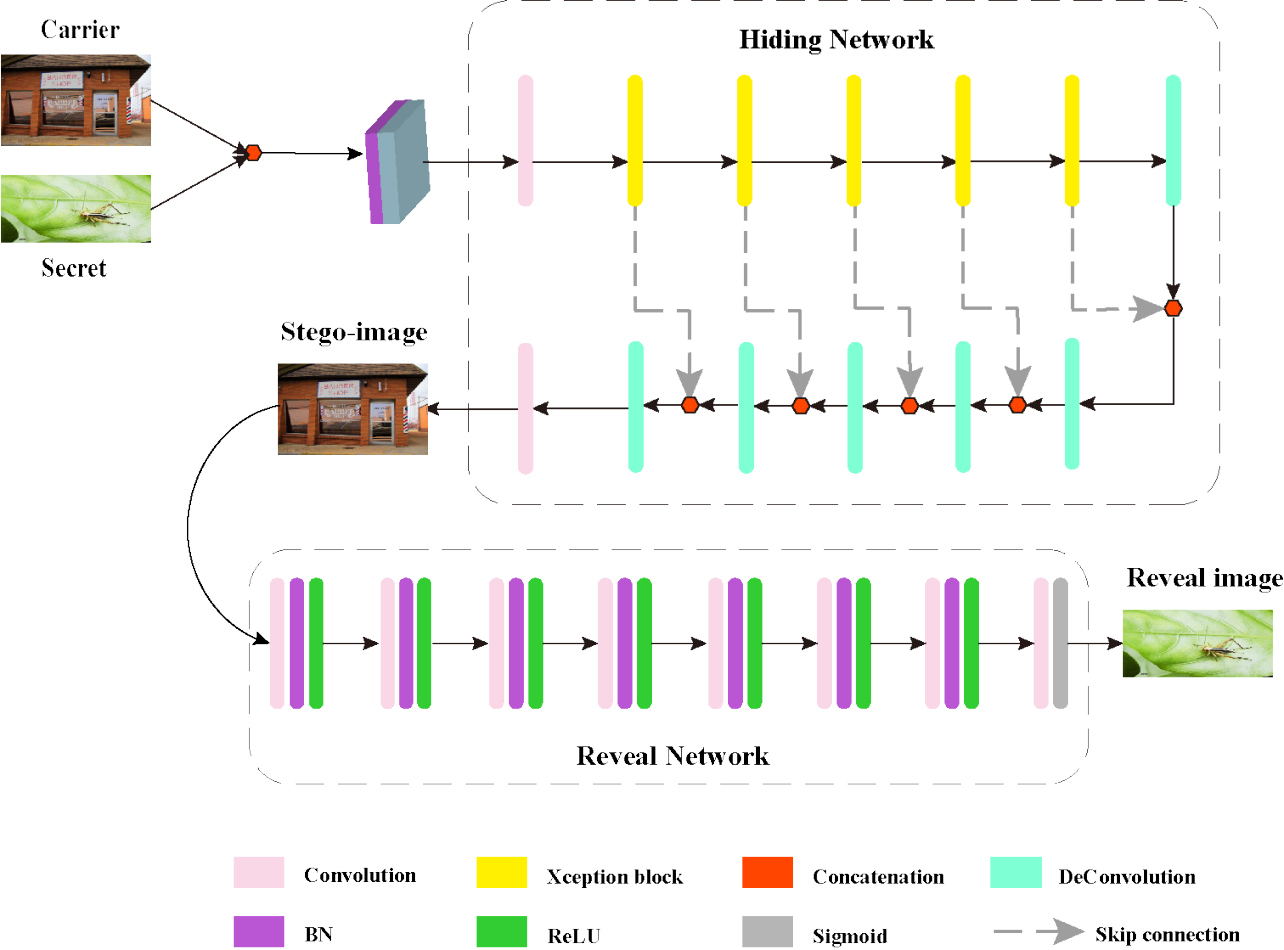
The proposed hidden framework.

Our proposed network first performs a cascading operation on the cover image (Cover) and the secret image (Secret), where the size and number of channels of the Cover and Secret are the same (the number of color image channels is 3, and the size is 256 × 256 ), and then through the hiding network (Hiding Network) to obtain the secret image (Stego-image), which is the image that we get after hiding, and then through the extraction network (RevSec Network) in order to reconstruct our hidden secret image (RevSec image). Figure 4 shows the concrete block diagram.

### 3.2. Hidden Network

**Figure 5:**
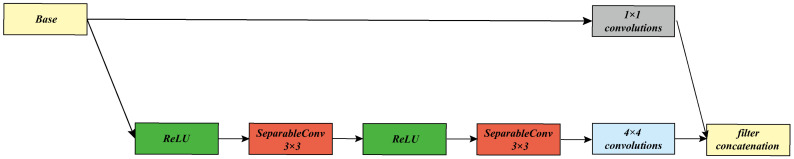
Xception block architecture.

The input of the hidden network is the image $R^{256\times 256\times 6}$ after the cascade of Cover and Secret, and the output is the hidden image $R^{256\times 256\times 3}$, which has two 4 × 4 convolutional layers and five Xception blocks (the Xception block is shown in Figure 5, as shown) and five residual blocks, of which the first 4 × 4 convolutional layer and five Xception blocks are used in order to reduce the feature dimension and allow for the secret image and cover image to perform feature fusion, and at the same time in the Xception Block the use of fully separable convolution in, can greatly reduce the parameters. The five residual blocks are composed of five deconvolutions and five concatenation operations. The cascade is to superimpose the two sets of feature maps that need to be cascaded. The function of the residual block is to restore the cover image and retain the characteristics of the secret image in the cover image. The function of the hidden network is to embed the secret image in each channel of the cover image and obtain a secret image that is similar to the cover image. Our goal is to minimize the loss between Cover and Stego-image during embedding:

(3) $$\tau =∥c-c^{\prime }∥$$

where *c* and $c^{\prime }$ represent the cover image and stego-image.

Where Figure 5 shows the architecture of the Xception block.

We will visualize the hiding and extraction operations in the proposed scheme in the form of pseudo code, as follows.
**Improved Xception Hidden and Extracted Procedures.****Input:***c*, *s*(1) Initialize the convolution operation weight: Normal(0.0,0.02);     Initialize the BatchNorm operation weight: Normal(1.0,0.02).(2)     x=Cat(c,s)(3)    **For**
*i* = 1 to 200 **do**(4)    $c^{\prime }=Encoder\left(x\right)$.(5)    Update the weight of the Encoder by $∥c-c^{\prime }∥$.(6)    $y=Decoder(c^{\prime })$.(7)    $s^{\prime }=RevealNetwork\left(y\right)$;(8)    Update the Encoder and Decoder weights through ζ.(9)    **End For****Output:**$c^{\prime }$, $s^{\prime }$

Table 1 shows the specific process of hidden network.

### 3.3. ExtractS Network

The input of the extraction network is the output of the hidden network. At the same time, the channel number and size of the hidden network will not change. As shown in Figure 4, the extraction network has eight convolutional layers, seven BN operations, seven ReLUs, and one Sigmoid function. The first six groups are composed of one convolutional layer, one BN operation, and one ReLU in order to form the last group. It consists of a convolutional layer and a Sigmoid function. Their main function is to reconstruct the secret image from the steganographic image that was obtained by the hidden network. Of course, our reconstructed RevSec image and the original secret image will have a certain loss. Our goal is to minimize this loss:

(4) $$\rho =∥s-s^{\prime }∥$$

Table 2 shows the specific process.

### 3.4. Loss Function

From the first two subsections, we can see that there are two losses in our network. The first is the loss τ of the Stego-image process that was obtained by the cascaded image through the hidden network, and the second is the Stego-image reconstruction through the extraction network The loss in the RevSec image process ρ. The total loss of the network can be expressed by the following formula:

(5) θ=τ+ρ

However, while minimizing the total loss θ, we should also pay attention not to cause too much distortion of the Stego-image or RevSec image, so we add a weight in order to maintain it so that can better hide and extract information, as follows:

(6) θ=τ+αρ

where α is the weight.

## 4. Experimental Results and Analysis

In this chapter, we introduce the specific experimental results and some data comparisons while using our proposed scheme. At the same time, we also tested the generalization ability and anti-stegal analysis ability of the scheme, and conducted ablation experiments in order to prove the rationality of our improved method. In this experiment, 20,000 images are used for model training, and 3000 images are used for model testing. These images are all from ImageNet. The initial learning rate of the model is 0.001, the hyperparameter α = 0.75, the batch of images entering the model is 8, and the number of training iterations is 200. In this experiment, the GPU that we used is NVIDIA GeForce 2070 (NVIDIA, Santa Clara, CA, USA), the experimental environment is Pytorch 1.1.0, the application is Python 3.6 for simulation experiments, and the number of GPUs is 2. Finally, we performed the visual subjective evaluation and SSIM and PSNR objective evaluation on the final results. At the same time, we will also conduct an ablation experiment in order to show the importance of our improvement to the original Xception network.

### 4.1. Visual Subjective Assessment

**Figure 6:**
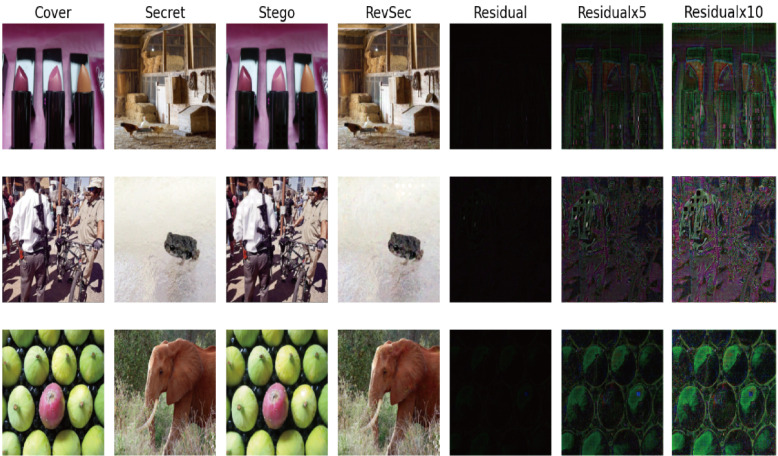
Randomly select three sets of images for subjective visual assessment.

First, the cover picture and secret picture (secret picture) were visually subjectively evaluated, and the reconstructed image, and also the error map between the cover image and the secret image. From Figure 6 (from left to right are cover image, secret image, stego image, revsec image, error diagram of cover image, and secret image (×1, ×5, ×10)), you can see the cover image and secret image. There is no visual difference between the secret image and reconstructed image. At the same time, we can only see the cover image artifacts when the error map is ×5 and ×10, respectively. No artifacts of the secret image are visible.

### 4.2. PSNR, SSIM Objective Evaluation

**Figure 7:**
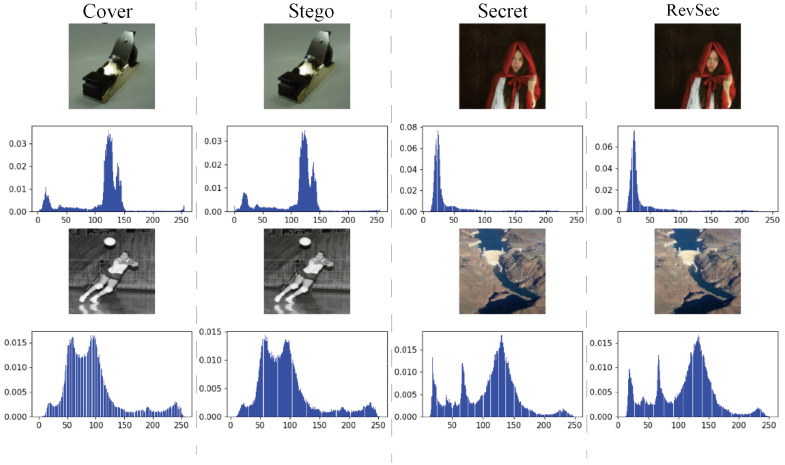
Random histogram test of two sets of experimental data.

PSNR is an important objective detection index of image quality, which is used in order to detect the distortion rate of the image. In this article, it is the logarithm of the mean square error between the cover image and the secret image, between the secret image and the reconstructed image, relative to ${\left(2^{n}-1\right)}^{2}$ (the square of the maximum signal). For example, given two images *A* and *B* of size (w, h), the mean square error (MSE) is:

(7) $$\mathrm{MSE}=\frac{1}{WH}\sum_{a=1}^{W}{\sum_{b=1}^{H}\left(A_{a,b}-B_{a,b}\right)}^{2}$$

where *n* is the number of bits in each sample value. SSIM is an important indicator of image similarity. The structural similarity (SSIM) between the cover picture and secret picture (SSIM) will be given, which is, given the cover image and the secret image *A* and *B*, the structural similarity can be calculated according to the following formula:

(8) $$\mathrm{SSIM}=\frac{\left(2\mu_{A}\mu_{B}+c_{1}\right)\left(2\sigma_{AB}+c_{2}\right)}{\left(\mu_{A}^{2}+\mu_{B}^{2}+c_{1}\right)\left(\sigma_{A}^{2}+\sigma_{B}^{2}+c_{2}\right)}$$

where $\mu_{A}$ is the average value of *A*, $\mu_{B}$ is the average value of *B*, $\sigma_{A}^{2}$ is the variance of *A*, $\sigma_{B}^{2}$ is the variance of *B*, $\sigma_{AB}$ is the covariance of *A* and *B*, $c_{1}={\left(k_{1}L\right)}^{2}$, $c_{1}={\left(k_{2}L\right)}^{2}$ is a constant used to maintain stability, and *L* is the range of pixel values. $k_{1}$ = 0.01, $k_{2}$ = 0.03. The value range is between 0–1. When the SSIM value is equal to 1, it means that the two images are exactly the same. At the same time, we randomly selected and drew the cover image and secret image. Figure 7 shows the histogram of the secret image and the reconstructed image, where, from left to right, are the cover image, the secret image, the stego image, the revsec image, and its histogram, and we can see that there is not much difference between them.

The two groups of randomly selected experimental results in Figure 7 and the average PSNR and SSIM of the scheme are shown in Table 3.

At the same time, we compared SSIM and PSNR with other solutions, and the results are shown in the Table 4.

### 4.3. Steganographic Capacity and Load Capacity

Steganographic capacity is an important indicator of network performance, which refers to the ratio of the size of the actual embedded secret information to the size of the cover information, which is Formula (10). In the case of the same image quality, the larger the steganographic capacity, the better.

(9) $$\mathrm{Relative}\;\mathrm{capacity}=\frac{\mathrm{Absolute}\;\mathrm{capacity}}{\mathrm{The}\;\mathrm{size}\;\mathrm{of}\;\mathrm{image}}$$

where absolute capacity refers to the size of the storage space that is occupied by the secret information or the size of the secret image. The size of image refers to the size of the cover image. Because the size of the secret image in our proposed scheme is equal to the size of the cover image, the relative capacity is 1. The steganographic capacity comparison results of the proposed scheme and other schemes are shown in Table 5.

The load capacity is the number of bits of information hidden by each pixel in the cover image. The load capacity can be obtained by the following formula:

(10) C=ω×8×3(bpp)

where *C* is the load capacity, each pixel occupies eight bits, a total of three channels, ω is the probability that we reconstruct the network to correctly reconstruct the secret image, which is obtained by the following formula:

(11) $$\omega =1-\frac{\sum_{a=1}^{W}\sum_{b=1}^{H}\left|S_{a,b}-R_{a,b}\right|}{W\times H}$$

**Figure 8:**
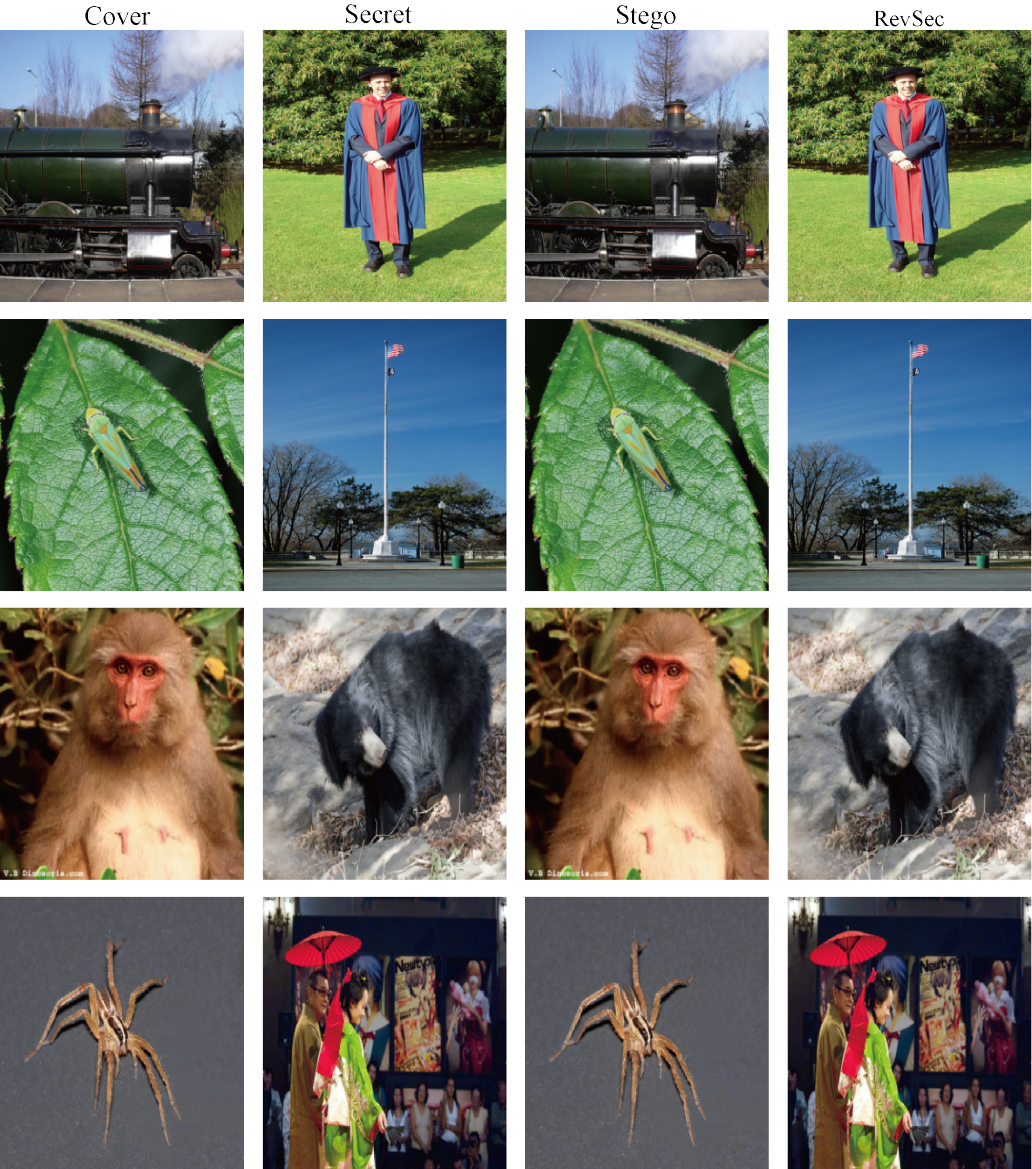
Four sets of experimental results randomly drawn for load capacity analysis.

We randomly selected four sets of experimental data to calculate the load capacity, as shown in Figure 8,the calculated load capacity is shown in Table 6 and, from left to right, are cover image, secret image, stego image, and revsec image. At the same time, 200 sets of experimental data are randomly selected to calculate its load capacity, and it can be concluded that the average load capacity of the proposed scheme is 23.96 bpp.

### 4.4. Generalization Ability Test

**Figure 9:**
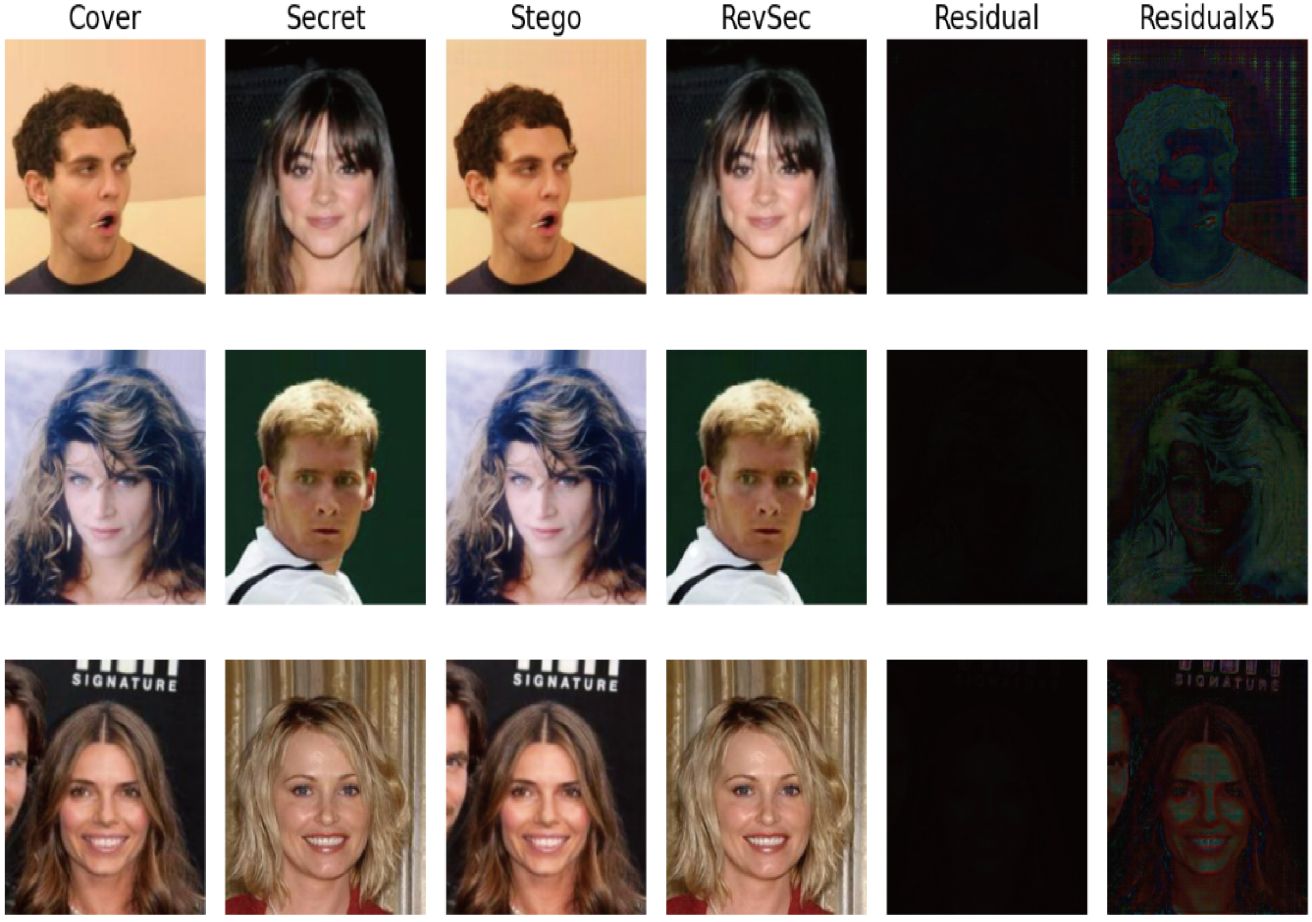
Test results of our proposed scheme on CelebA.

**Figure 10:**
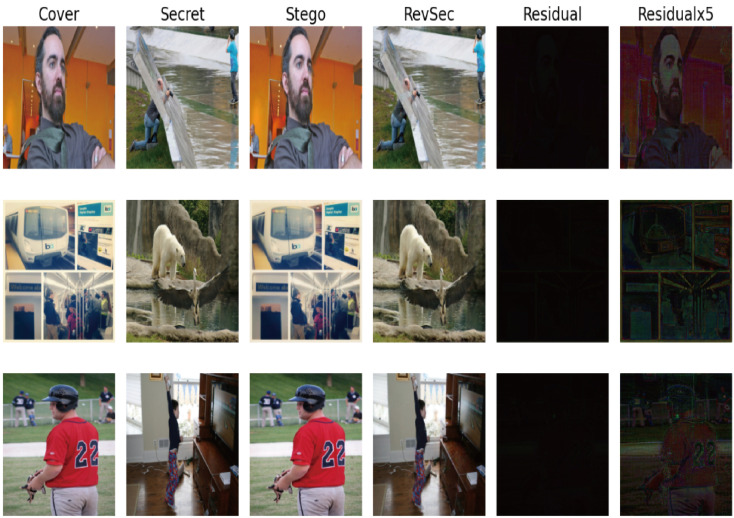
Test results of our proposed scheme on COCO.

**Figure 11:**
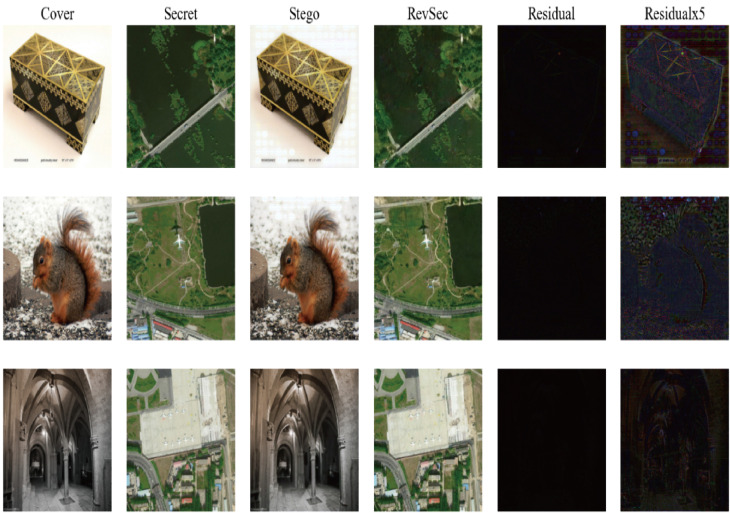
The test results of our proposed scheme on aerial images.

**Figure 12:**
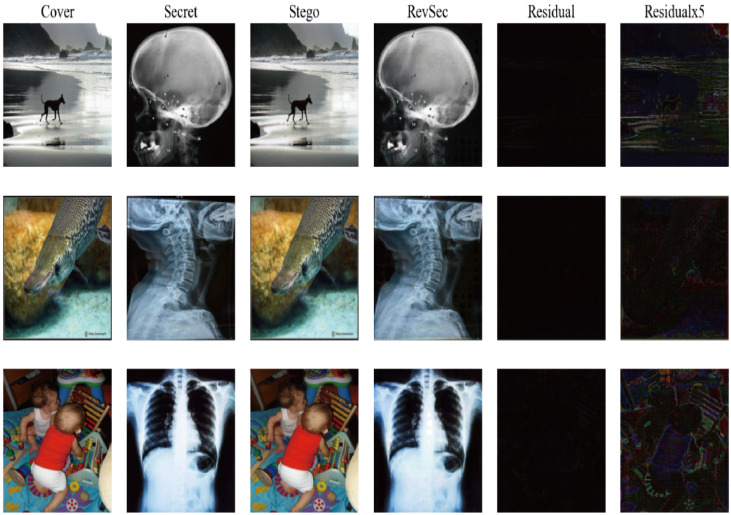
The test results of our proposed scheme on CT images.

Generalization ability refers to the adaptability of the program in new samples. The purpose of learning is also to learn the laws behind the data. Other data sets with the same laws can also get appropriate output through the network [26]. For image steganography, using samples other than the training set can also achieve the same steganography effect without reducing PSNR and SSIM. In order to ensure the generalization ability of our proposed model, we randomly selected some images from other data sets for testing, such as CelebA (Figure 9) and COCO (Figure 10); at the same time, we have also tested application scenarios, such as aerial images (Figure 11) and CT images (Figure 12). The results prove that our proposed model works well. The result images of each of the following tests are the cover image, the secret image, the secret image, the reconstructed image, the error map of the cover image, and the secret image (×1, ×5) from left to right.

### 4.5. Ablation Experiment

**Figure 13:**
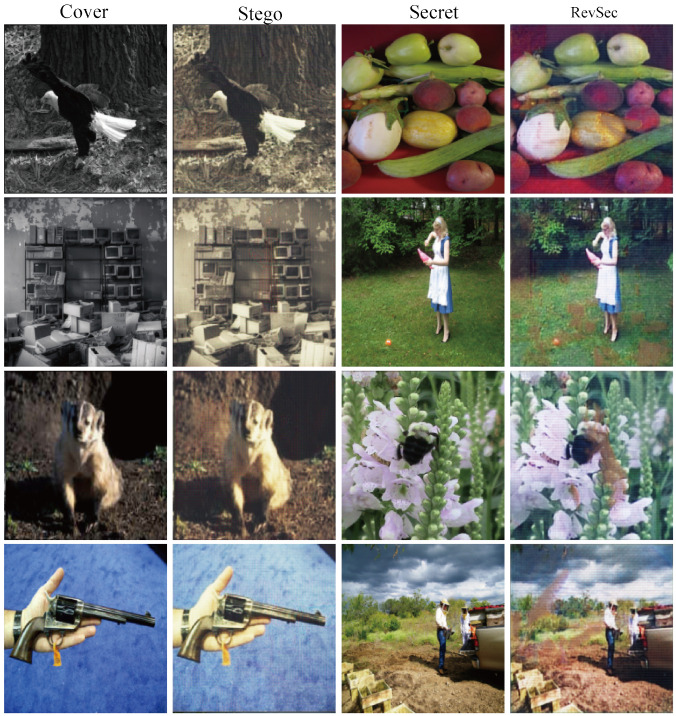
Results of ablation experiment after 158 iterations.

The ablation experiment is to prove the advantages of changing the model. Here, in order to prove the importance of the jump connection, we added whether we removed the jump connection and did this experiment again, and the result is shown in Figure 13, and, from left to right, are the cover image, secret image, stego image, and revsec image. This is the training image obtained after 158 iterations. It can be seen that the image convergence effect is still not satisfactory.

### 4.6. Steganalysis Test

**Figure 14:**
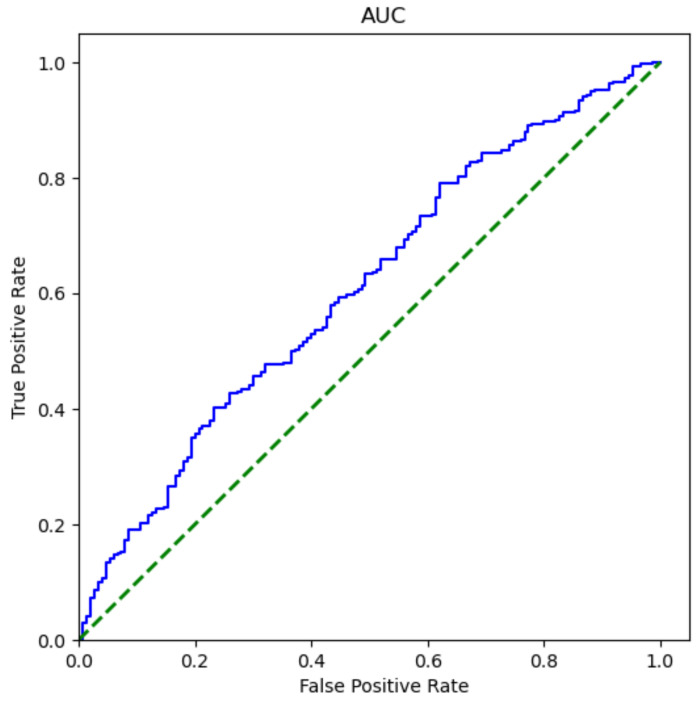
ROC curve of steganalysis.

Steganalysis is a means to detect whether there is hidden information in an image. Here, in order to test the performance of our experimental results against steganalysis, the steganalysis tool StegExpose [27] has been used for steganalysis. In the experiment, we used a standard threshold with a threshold of 0.2 and drew the ROC curve, as shown in Figure 14. The green line represents the random prediction, and the blue line represents the prediction of the steganalysis tool. You can see that the result of using the steganalysis tool is closer to the random prediction result.

## 5. Conclusions

In this article, we use deep learning instead of manual design in order to design the cost function. For the first time, we used the Xception network to hide the secret image with the same size as the cover image and improve it. Jump connections have been added to the Xcption network to greatly speed up the convergence speed of the network. The experiments show that this scheme has a high steganography capability, with a load capacity of 23.96 bpp, which can realize high-capacity image steganography. Stego images and RevSec images also have high PSNR and SSIM, and the average PSNR and SSIM are higher than other existing image steganography schemes. At the same time, it has good generalization ability. When testing on other data sets, you can obtain the same effect as the training set. However, this solution also has some limitations. For example, when we use a high-texture image as a secret image and a low-texture image as a cover image, the result will be worse than the normal image. In the next step, we will introduce an attention module in order to improve the model, hide the secret image more in the high-texture area of the cover image, to obtain higher SSIM and PSNR, and introduce an encryption mechanism, when using steganalysis algorithms to know that we may have hidden information, it cannot get a meaningful real image, only an encrypted image. Finally, as attacks on stego images, such as cropping, compression, etc., will cause our secret images to be unable to be reconstructed, in the next step we will add cropping, compression, and other operations to the model in order to improve the model’s anti-attack ability through adversarial training.

## Figures and Tables

**Table 1:** Architecture of hidden networks.

Process	Input (Channal)	Output (Channal)	Size (H × W)
Conv (k = 4, s = 2, p = 1)	6	32	128 × 128
Xception block	32	64	64 × 64
Xception block	64	128	32 × 32
Xception block	128	256	16 × 16
Xception block	256	512	8 × 8
Xception block	512	1024	4 × 4
DeConv (k = 4, s = 2, p = 1)	1024	512	8 × 8
DeConv (k = 4, s = 2, p = 1)	1024 + 512	512	16 × 16
DeConv (k = 4, s = 2, p = 1)	512 + 256	256	32 × 32
DeConv (k = 4, s = 2, p = 1)	256 + 128	128	64 × 64
DeConv (k = 4, s = 2, p = 1)	128 + 64	64	128 × 128
DeConv (k = 4, s = 2, p = 1)	64 + 32	32	256 × 256
Conv (k = 3, s = 1, p = 1)	32	3	256 × 256

**Table 2:** Architechure of RevSec network.

Process	Output Size (H × W × D)
Conv (k = 3, s = 1, p = 1) + BN + ReLU	256 × 256 × 32
Conv (k = 3, s = 1, p = 1) + BN + ReLU	256 × 256 × 64
Conv (k = 3, s = 1, p = 1) + BN + ReLU	256 × 256 × 128
Conv (k = 3, s = 1, p = 1) + BN + ReLU	256 × 256 × 256
Conv (k = 3, s = 1, p = 1) + BN + ReLU	256 × 256 × 128
Conv (k = 3, s = 1, p = 1) + BN + ReLU	256 × 256 × 64
Conv (k = 3, s = 1, p = 1) + BN + ReLU	256 × 256 × 32
Conv (k = 3, s = 1, p = 1) + Sigmoid	256 × 256 × 3

**Table 3:** Peak signal-to-noise ratio (PSNR) and structural similarity (SSIM) of stego-image and secret image, secret image, and RevSec image.

	Cover and Stego-Image	Secret vs. RevSec
Figure	(PSNR, SSIM)	(PSNR, SSIM)
Figure 7 row1	40.632, 0.997	37.433, 0.979
Figure 7 row3	40.327, 0.995	37.021, 0.971
Average	40.211, 0.993	37.704, 0.983

**Table 4:** Comparison with other methods.

	Cover and Stego-Image	Secret vs. RevSec
Schemes	(PSNR, SSIM)	(PSNR, SSIM)
[19]	34.892, 0.968	33.420, 0.947
[20]	32.907, 0.960	36.601, 0.960
[13]	39.894, 0.987	37.455, 0.977
Average	40.211, 0.993	37.704, 0.983

**Table 5:** Steganographic capacity comparison.

	Absolute Capacity	Stego-Image Size	Relative Capacity
Schemes	(Bytes or Pixel)	(Pixel)	(Bytes/Pixel)
[21]	≥37.5	64 × 64	$9.16\times 10^{-3}$
[22]	0.375	32 × 32	$3.7\times 10^{-4}$
[23]	26,214∼104,857	512 × 512	$1\times 10^{-1}$∼$4\times 10^{-1}$
[24]	1535∼4300	1024 × 1024	$1.46\times 10^{-3}$∼$4.10\times 10^{-3}$
[25]	18.3∼135.4	64 × 64	$1.49\times 10^{-3}$∼$1.10\times 10^{-2}$
Ours	256 × 256	256 × 256	1

**Table 6:** Load capacity of randomly selected four sets of samples.

Figure	Load Capacity (bpp)
Figure 8 row1	23.96
Figure 8 row2	23.96
Figure 8 row3	23.95
Figure 8 row4	23.97
